# Isolation and identification of *Streptococcus suis* from sick pigs in Bali, Indonesia

**DOI:** 10.1186/s13104-019-4826-7

**Published:** 2019-12-05

**Authors:** I Nengah Kerta Besung, I Gusti Ketut Suarjana, Kadek Karang Agustina, Ida Bagus Oka Winaya, Hamong Soeharsono, Ni Ketut Suwiti, Gusti Ngurah Mahardika

**Affiliations:** 1 Laboratory of Microbiology, Faculty of Veterinary Medicine, Udayana University of Bali, Jl. Sudirman, Denpasar, 80225 Indonesia; 2 Department of Veterinary Public Health, Faculty of Veterinary Medicine, Udayana University of Bali, Jl. Sudirman, Denpasar, 80225 Indonesia; 3 Laboratory of Pathology, Faculty of Veterinary Medicine, Udayana University of Bali, Jl. Sudirman, Denpasar, 80225 Indonesia; 4 Laboratory of Biochemistry, Faculty of Veterinary Medicine, Udayana University of Bali, Jl. Sudirman, Denpasar, 80225 Indonesia; 5 Department of Basic Veterinary Science, Faculty of Veterinary Medicine, Udayana University of Bali, Jl. Sudirman, Denpasar, 80225 Indonesia; 6 The Animal Biomedical and Molecular Biology Laboratory, Udayana University of Bali, Jl. Sesetan-Markisa #6, Denpasar, 80226 Indonesia

**Keywords:** Glutamate dehydrogenase (GDH) gene, Recombination/repair protein (recN) gene, *Streptococcus suis*, Sick pigs, Bali, Indonesia

## Abstract

**Objective:**

*Streptococcus suis* (*S. suis*) is a causative agent for various syndromes in pigs. It can be transmitted to humans with typical symptoms of meningitis and death. Although human infections have been confirmed at Bali Referral Hospital, Indonesia, since 2014, the bacteria have not been isolated from pigs. Here, we provide confirmation of the presence of the bacteria in sick pigs in the province.

**Results:**

*Streptococcus suis* was confirmed in 8 of 30 cases. The final confirmation was made using PCR and sequencing of the glutamate dehydrogenase (GDH) and recombination/repair protein (recN) gene fragments. Upon PCR serotyping, two were confirmed to be serotype 2 or 1/2. Prominent histopathological lesions of confirmed cases were meningitis, endocarditis, pericarditis, bronchopneumonia, enteritis and glomerulonephritis. The dominant inflammatory cells were neutrophils and macrophages. Further research is needed to understand the risk factors for human infection. Community awareness on the risk of contracting *S. suis* and vaccine development are needed to prevent human infections.

## Introduction

*Streptococcus suis* (*S. suis*) is an important agent of emerging zoonotic, community-acquired bacterial meningitis [1–3]. It is a global zoonotic agent originating in pigs [4]. Asia is a unique hotspot of *S. suis* infection in humans related to traditional pork consumption customs [5, 6]. The bacteria cause significant economic losses through mortality and decreased production [7–9] and indirect losses through decreased demand for pork if an outbreak occurs.

This emerging zoonotic bacterium is of particular concern in tourist destinations because it poses an infection risk to travelers and local people. Bali Province in Indonesia is one of world’s most prominent tourist destinations. More than six million travelers visited Bali in 2018 (https://bali.bps.go.id). Data from Bali Referral Hospital showed more than 40 confirmed human cases of *S. suis* meningitis since 2014 [10]. Cases of *S. suis* infection in pigs with encephalitis and arthritis signs have long been suspected in the province. Attempts to detect the bacteria have not yet been successful. This is likely because of identification problems with *S. suis*. Its growth characteristic of forming small colonies [11] means that this bacterium might be overlooked in a culture with mixed bacterial species. Misidentification of *S. suis* is also common [12–14]. The false negative result can be solved through a complete serotyping system [15], which is not available in Indonesia.

## Main text

### Methods

#### Source of animals

Sick pigs were owned by individual farmers, who contacted The Animal Biomedical and Molecular Biology Laboratory of Udayana University reporting sickness and mortality in their piggery. They agreed for their sick and dead animals to be included in the study. The farms were located across Bali Province (Table 1).

**Table 1:** Clinical signs, animal age, number of animal in stall of confirmed *S. suis* infection in Bali, Indonesia, 2018

No	Location (village and regency)	Number of animal in stall	Number of sick animals	Age (month)	Number of death	Clinical signs
1	Abang, Karangasem	3	2	8	1	In-appetence, depression, reddish skin discoloration, cough
2	Sesetan, Denpasar	100	21	4	3	Fever, anorexia, lethargic, limping, paralyzed, tremor, eye and nasal exudate, erythema ear, joint enlargement and bruising
3	Payangan, Gianyar	4	1	8	5	Weakness, anorectic, diarrhea, watery nose, eye exudate, reddish skin discoloration, limping
4	Kesiman Denpasar	8	6	10	6	Diarrhea, limping, anorexia, paralyzed, snot
5	Sidakarya, Denpasar	150	14	3	3	Yellowish diarrhea, dyspnea, nasal exudate, shivering, reddish skin discoloration
6	Kediri, Tabanan	68	11	7	3	Weakness, anorexia, diarrhea, nasal and eye exudate, limping, reddish skin discoloration
7	Perean Kangin, Tabanan	12	7	6	3	Lethargic, anorexia, reddish skin discoloration, shivering
8	Lod tunduh, Gianyar	24	7	12	7	Anorexia, lethargic, cough, reddish skin discoloration, swollen joint

During January to July 2018, 30 suspected *S. suis* cases were recorded. The inclusion criteria were acute illness with at least one clinical sign of neurological disorder, reddish skin discoloration and arthritis. The animals were not treated with antibiotics.

Freshly dead animals were necropsied. Organs with clear pathological lesions were collected in Stuart Transport Medium (CM0111 Oxoid) and buffered formalin. Isolation and biochemical characterization were conducted according standard protocol [16]. The tissues from one animal were pooled and extracted. The suspension was plated on a 5% defibrinated sheep blood agar plate. The plate was incubated at 37 °C for 18–24 h. Some suspected colonies were Gram stained and grown in triple sugar iron agar and sulfide indole motility media. Other tests were catalase, oxidase, citrate, methyl red, Voges–Proskauer (VP), glucose and lactose tests. Three suspected colonies were injected to separate tryptic soy broth (Sigma Aldrich MFCD00132536) and incubated at 37 °C for 18–24 h. DNA was isolated using 10% chelex-100 (Biorad, CA) [17, 18]. The glutamate dehydrogenase (GDH) and recombination/repair protein (recN) gene fragments were amplified using polymerase chain reaction (PCR) using published specific primer sets for *S. suis* [19, 20]. The GDH and recN fragments were sequenced by Apical Scientific Sequencing (Malaysia) using an automatic chain termination method. Serotyping was conducted using PCR to detect the *cps1I* gene for serotype 1 and 14 and the *cps2I* gene for 2 and 1/2 as recommended [21].

Tissue was processed and stained with hematoxylin and eosin (H&E) staining based on a published protocol [22].

### Results

Out of 30 cases, 8 were indicative for *S. suis* and showed small and non-hemolytic colonies that were Gram positive with coccus-chain forming appearance. Only positive cases are described further in this manuscript. The epidemiological and clinical data of the presumably positive cases are presented in Table 1. Listed from the most frequent, clinical signs were reddish skin discoloration, anorexia, nasal exudate, diarrhea, limping, eye exudate, lethargy, swollen joints, shivering, weakness, cough, lack of appetite, depression, dyspnea, tremor, fever and snot. Animal were aged 3–12 months. The cases were from Denpasar, Gianyar, Tabanan and Karangasem regencies.

The results of Gram staining and biochemical tests of suspected colonies from the eight suspected *S. suis* infections are presented in Table 2. All isolates were Gram (+), coccus form, short chained and positive in acid slant, catalase and lactose, but negative in VP test.

**Table 2:** Gram staining and biochemical properties of bacterial colonies of confirmed *S. suis* infection in Bali, Indonesia

No	Microscopic after Gram staining	TSIA				SIM			Catalase	VP	Lactose
		Acid slant	Acid butt	Gas	H_2_S	H_2_S	Indole	Motile			
1	Gram positive, coccus, short chain	+	−	−	−	−	−	−	+	−	+
2	Gram positive, coccus no chain	+	−	−	−	−	−	−	+	−	+
3	Gram positive, coccus, no chain	+	−	−	−	−	−	−	+	−	+
4	Gram positive, coccus, no chain	+	−	−	−	−	−	−	+	−	+
5	Gram positive, coccus, no chain	+	−	−	−	−	−	−	+	−	+
6	Gram positive, coccus, no chain	+	−	−	−	−	−	−	+	−	+
7	Gram positive, coccus, short chain	+	−	−	−	−	−	−	+	−	+
8	Gram positive, coccus, no chain	+	−	−	−	−	−	−	+	−	+

The results of oxidase, citrate, MR, and glucose are not shown; all was negative

After electrophoresis of PCR products (not shown), positive samples showed a single band of around 700 bp for GDH and 350 bp for recN. The GDH sequences of five isolates of *S. suis* from our study were identical. The blast result showed a percentage identity (PID) of 99.09% to human isolates from Bali Indonesia as published previously [10]. The sequences of recN of two isolates show a PID of 100% to human isolates, while other two each have 98.5%. Upon PCR serotyping, two isolates were confirmed to be serotype 2 or 1/2. Two *cps2I* sequences of animal isolates in our study are completely identical to those of human isolates [10]. The GenBank Acc. No. of GDH, recN and *cps2I* for serotype 2 or 1/2 are MN334770–MN334774, MN520294–MN520297, and MN520292–MN520293, respectively.

**Fig. 1:**
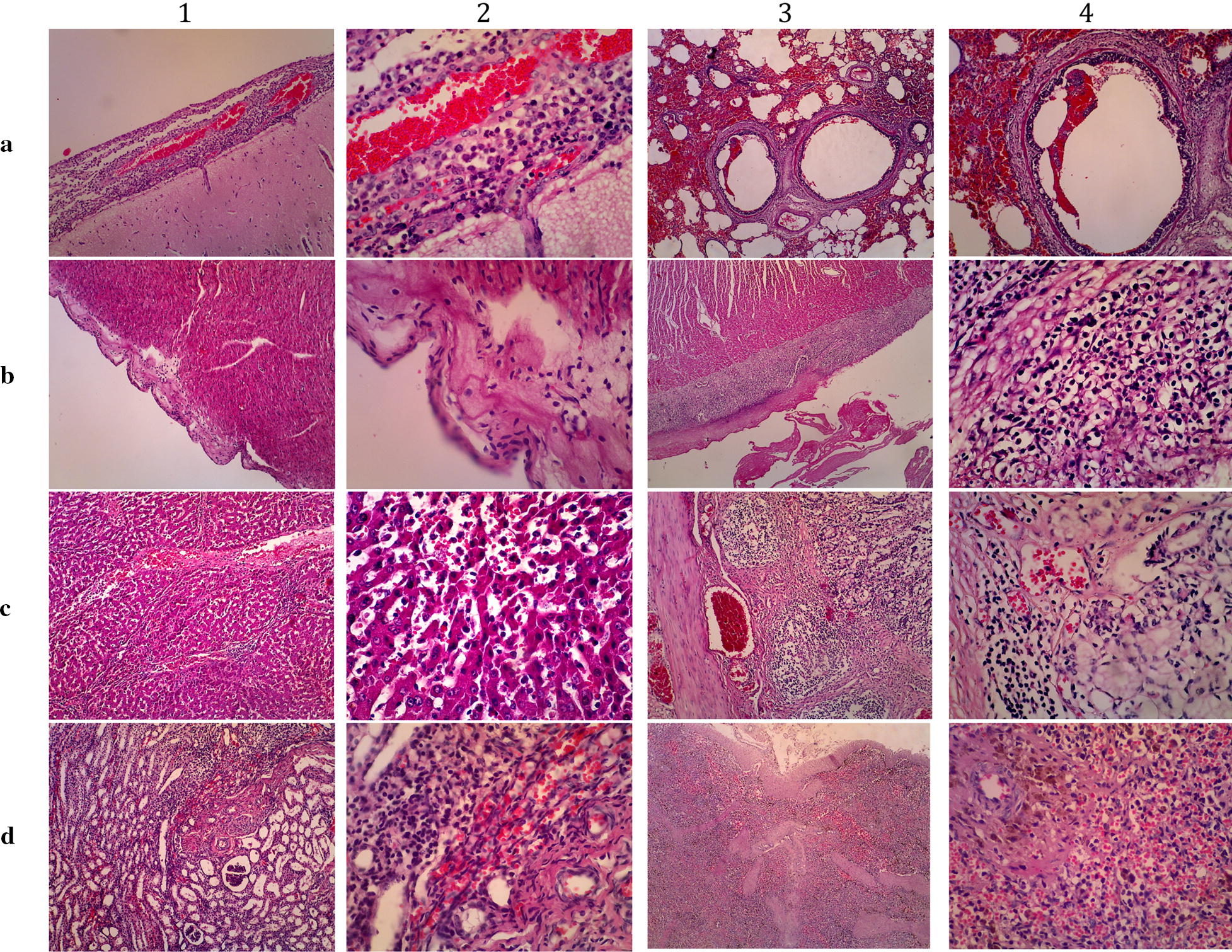
Histopathological pictures of various tissues of confirmed *S. suis* infection in pig cases in Bali, Indonesia, 2018. Panel **a1**–**2** are brain and meninges showing meningitis; **a3**–**4** are lung tissues showing bronchopneumonia; **b1**–**2** are myocardium showing endocarditis; **b3**–**4** are myocardium showing pericarditis; **c1**–**2** are liver showing congestion and hepatitis; **c3**–**4** are intestine showing hemorrhagic enteritis with depletion of Payer patches; **d1**–**2** are kidney showing hemorrhagic glomerulonephritis; **d3**–**4** are spleen showing perifollicular infiltration of inflammatory cells and hemorrhage; H&E stained. Magnifications in column 1 and 3 are ×100; Column 2 and 4 are ×400

A panel of histological pictures of confirmed cases is depicted in Fig. 1. The histopathologies of confirmed cases were similar with varying severities. The prominent pictures were congestion in the brain, meningitis, bronchopneumonia, endocarditis, myocarditis, pericarditis, erosion and enteritis along the gastrointestinal tract with obvious depletion of Payer patches, hemorrhagic hepatitis and glomerulonephritis, as well as lymphoid depletion, hemorrhage and accumulation of inflammatory cells in the spleen. Dominant inflammatory cell infiltration in those tissues was neutrophil and macrophage.

### Discussion

We confirmed that *S. suis* does present and cause illness in pigs in Bali. Human cases confirmed at Bali Referral Hospital [10] must have originated from pigs. *Streptococcus suis* meningitis is a global zoonotic community-acquired bacterial meningitis [1, 2, 4]. The bacterium is of extra importance in Asia as human outbreaks are related to traditional pork consumption practices [5, 6]. The practice of consuming a delicacy of raw pork with raw blood is also common in Bali.

In this study, we carefully selected suspected cases, especially those of acute cases with no history of antibiotic medication. The cases occurred in pigs under 1 year old. Although *S. suis* can be isolated from sick and healthy pigs at various ages [23], clinical manifestation seems more frequent in young animals [24]. A study in Canada [25] showed that *S. suis* was more frequently isolated from pigs aged between 5 and 10 weeks.

We recorded clinical signs in our cases involving many organs of central nervous, respiratory, urogenital and circulatory systems and gastrointestinal tract. Recorded clinical signs of *S. suis* infection in the literature can indeed be multi-organ. The signs can be pyrexia, lack of appetite, depression, nasal discharge, dyspnea, tremors, seizures, incoordination, unusual stances (such as sitting like a dog), inability to stand, paddling, opisthotonos, convulsions, nystagmus, skin disease, swollen limbs and death [26]. In some cases, the disease goes per-acute and ends with sudden death without obvious signs [26]. Although septicemia and meningitis are the most striking manifestations of the disease, endocarditis, pneumonia and arthritis have been reported [27]. Another review article also reported that the disease syndromes caused by *S. suis* in swine include arthritis, meningitis, pneumonia, septicemia, endocarditis, polyserositis, abortion and abscesses [28]. In a recent experimental infection, affected pigs presented clinical signs of anorexia, depression, fever, glazed eyes, reddened mucous membranes, severe nervous symptoms (incoordination, lateral prostration, paddling, opisthotonos, convulsions and lameness in the posterior limbs) [29].

Our suspected cases were from all regencies in Bali Province. However, the confirmed cases were from four regencies, namely Tabanan, Denpasar, Gianyar and Karangasem. Considering that Bali is a small island of 5.600 km^2^ with a high population density (http://www.baliprov.go.id/v1/geographi) and understanding the free movement of animals in the province, we assume that *S. suis* is distributed throughout the province. The morbidity, mortality and case fatality rates in our study were 18.7%, 8.4% and 44.9%, respectively. Morbidity, mortality and case fatality rates of *S. suis* in pigs vary [30]. Therefore, we assumed that our observation on the case epidemiology is plausible.

Our microbiological data confirmed *S. suis*. Gram staining, chain formation and biochemical characterization shows that identified isolates were Gram (+), coccus with grape-like or short chain. All were acid slant, catalase and lactose positive, while VP test was negative. *S. suis* is an encapsulated gram-positive bacterial coccus that occurs singly, frequently in pairs, or occasionally in short chains [3]. Most strains are alpha-hemolytic on bovine and sheep blood agar plates after 24 h of incubation at 37 °C [3]. Four tests are used for a presumptive identification of *S. suis*, that is, no growth in 6.5% NaCl agar, a negative VP test and production of acid in trehalose and salicin broths [25, 31]. The VP test is critical in differentiating *S. suis* from other *Streptococcus* species [15].

Final confirmation was made using PCR of GDH and recN. Both gene fragments are proposed as a system for reclassifying *S. suis* or as a specific PCR system for *S. suis* [19, 20]. We have established the system for *S. suis* detection from human and animal samples at Udayana University, Bali. The GDH blast result showed a PID of 99.09%, while four recN sequences showed PIDs of 98.5% and 100% to human isolates from Bal [10]. Upon PCR serotyping, two isolates were positive with primer sets for serotype 2 or 1/2 but not those of serotype 1 and 14. Only two primer sets were available in our lab. The readable sequences were identical to the *S. suis* serotype 2 and 1/2 *Cps2I* gene Ref. [21].

Although we carefully selected the cases with inclusion criteria of suspected cases that were acute and had at least one clinical signs of neurological disorder, reddish skin discoloration and arthritis, as well as no history of antibiotic treatment, only 8 of 30 suspected cases were confirmed as *S. suis* infection. The animals were not treated with antibiotics. The negative cases might have been caused by other infectious agents such as *Haemophilus parasuis*, pseudorabies or *Escherichia coli* [32].

Prominent histologic pictures were congestion in the brain and meningitis, bronchopneumonia, myocarditis, erosion and enteritis along the gastrointestinal tract, hemorrhagic liver and kidney. Dominant inflammatory cell infiltration in those tissues was neutrophilic. Gross and microscopic findings of *S. suis* include one or more of fibrinous polyserositis, fibrinous or hemorrhagic bronchopneumonia, purulent meningitis, myocardial necrosis, focal myocarditis and valvular endocarditis [30]. Moreover, meningoencephalitis, a striking lesion, has been found in China [29]. Meningitis histology in one case (Fig. 1, panel A1–2) resembles meningitis as described by that group.

This study was the result of passive surveillance in which the owners contacted our laboratory reporting sickness and mortality in their piggeries. The study did not investigate the link of animal cases to human infections. Active surveillance should be conducted to determine the overall risk factors and elucidate the link between animal isolates and human cases. That the GDH sequences of animal isolates are not identical to those of human isolates and the variation of recN and the confirmed serotype of 2 or 1/2 in two out of eight isolates expands our knowledge that the circulating bacteria might vary in the province. This should be mapped as it will benefit understanding of human transmission.

In conclusion, *Streptococcus suis* has been confirmed in sick pigs in Bali, Indonesia. Two isolates were confirmed to be serotype 2 or 1/2. Further research is needed to elucidate the risk factors for human infection and to map the distribution of *S. suis* in Indonesia. The fragment of GDH or recN might be used to map infections in Indonesia. Vaccines can be developed using inactivated strain [33] to reduce economic losses and the risk of human infection, including among domestic and international travelers.

## Limitations

The distribution of *S. suis* in Bali and Indonesia is not yet available.

## Data Availability

The GenBank Acc. No. of GDH, recN and *cps2I* for serotype 2 or 1/2 are MN334770–MN334774, MN520294–MN520297, and MN520292–MN520293, respectively. The sequence data are in the process to be made available in dryad (https://datadryad.org).

## Abbreviations

GDH: glutamate dehydrogenase
MR: methyl red
PCR: polymerase chain reaction
recN: recombination/repair protein
SIM: sulfide indole motility
TSB: tryptic soy broth
TSIA: triple sugar iron agar
VP: Voges–Proskauer
